# Game of Cruxes: co-designing a game for scientists and stakeholders for identifying joint problems

**DOI:** 10.1007/s11625-021-00983-2

**Published:** 2021-06-10

**Authors:** Nicolas Salliou, Enora Bruley, Clémence Moreau, Tobias Luthe, Victor Blanco, Sandra Lavorel, Adrienne Grêt-Regamey

**Affiliations:** 1 Department of Civil, Environmental and Geomatic Engineering, Institute for Spatial and Landscape Development, Planning of Landscape and Urban Systems (PLUS), ETH Zürich, Stefano-Franscini-Platz 5, CH-8093 Zürich, Switzerland; 2 Laboratoire d’Ecologie Alpine, CNRS, Université Grenoble Alpes, Université Savoie Mont Blanc, 38000 Grenoble, France; 3 SENS, IRD, CIRAD, Université Paul Valery Montpellier 3, University of Montpellier, Montpellier, France; 4 The Oslo School of Architecture and Design AHO, Maridalsveien 29, 0175 Oslo, Norway; 5 MonViso Institute, 12030 Ostana, CN Italy; 6 Institute of Science, Technology and Policy, ETH Zürich, Universitätstrasse 41, 8006 Zürich, Switzerland

**Keywords:** Participation, Serious game, Adaptation pathways, Mountain socio-ecological system

## Abstract

Scientists increasingly cross their disciplinary boundaries and connect with local stakeholders to jointly solve complex problems. Working with stakeholders means higher legitimacy and supports practical impact of research. Games provide a tool to achieve such transdisciplinary collaboration. In this paper, we explore the use of a game in a participatory project where scientists and local stakeholders are seeking and defining a joint problem. The literature is clear that this step is essential but remains short on concrete methods. Here, we explore this potential in practice. We conducted parallel participatory processes in two alpine regions considered as socio-ecological system (SES) in Switzerland and France, both vulnerable to global change. Based on these two case studies, we co-constructed a game, integrating scientific concerns about key land use, climate change and socio-economic elements of a mountain SES (tourism, agriculture, housing and demography). With the game, we assessed the existence of joint problems connecting scientific and local interests. The game successfully engaged participants at both sites over 11 game sessions, showing potential of use in other transdisciplinary settings. By covering a wide array of issues, the game created a discussion space for listing problems and identifying where scientist and stakeholder interests overlap. In Switzerland, the game revealed no pressing joint problem to be addressed. In France, game sessions revealed, among other problems, an enduring and complex issue regarding the co-existence of inhabitants and powerful institutions. Having demonstrated the capacity of this game for joint-problem assessment, we believe other participatory research in similar SES could benefit from an early use of such an approach to frame the potential for collaboration.

**Supplementary Information:**

The online version contains supplementary material available at 10.1007/s11625-021-00983-2.

## Introduction

Since Duke’s key book “Gaming: the future’s language” (Duke 1974), games are seen as one way to explore, learn and eventually solve complex and wicked problem. Games are particularly relevant to deal with uncertainty and a plurality of actor perspectives (Klabbers 1996). They have been used in education (Garcia et al. 2016; Wouters et al. 2009), natural resource management (Etienne 2013), urban planning (Poplin 2011), climate adaptation (Flood et al. 2018) and many other fields. The use of games may trigger efficient learning among a diversity of end-users, from students to stakeholders facing “real-life” issues. In science, games have been successfully used to tackle complex problems by engaging with gamers, like molecule folding (Cooper et al. 2010; Lee et al. 2014). Games can be used as a boundary object (Star and Griesemer 1989) to facilitate the discussion among a diversity of stakeholders and thus eventually enhance negotiation, coordination, cooperation or concertation (Etienne 2013).

A particular use of games implies the participation of stakeholders and experts in some of the steps of the modelling underlying the game construction: problem setting, conceptualization, design and simulations (Voinov and Bousquet 2010). Participation in this context may facilitate the integration of diverse perspectives, knowledge and issues into the modelled reality, thus improving the design and relevance of such models (Smetschka and Gaube 2020). It may also enhance the legitimacy of the game itself by ensuring an early connection to potential end-users. Additionally, participation in the game development appears to enhance the capacities of participants by expanding their understanding of the issue at stake, in particular by getting to know other stakeholders’ perspectives in more detail (Mathevet et al. 2011). In general, in democratic societies, such approaches may enhance the dialogue about debated and complex issues and lead to better decision-making, especially when stakes and uncertainties are high (Funtowicz and Ravetz 1994). In non-democratic societies, it may help to give voice to minorities and try to include them in decisions made by others but that impact them (Barnaud et al. 2010).

**Fig. 1:**
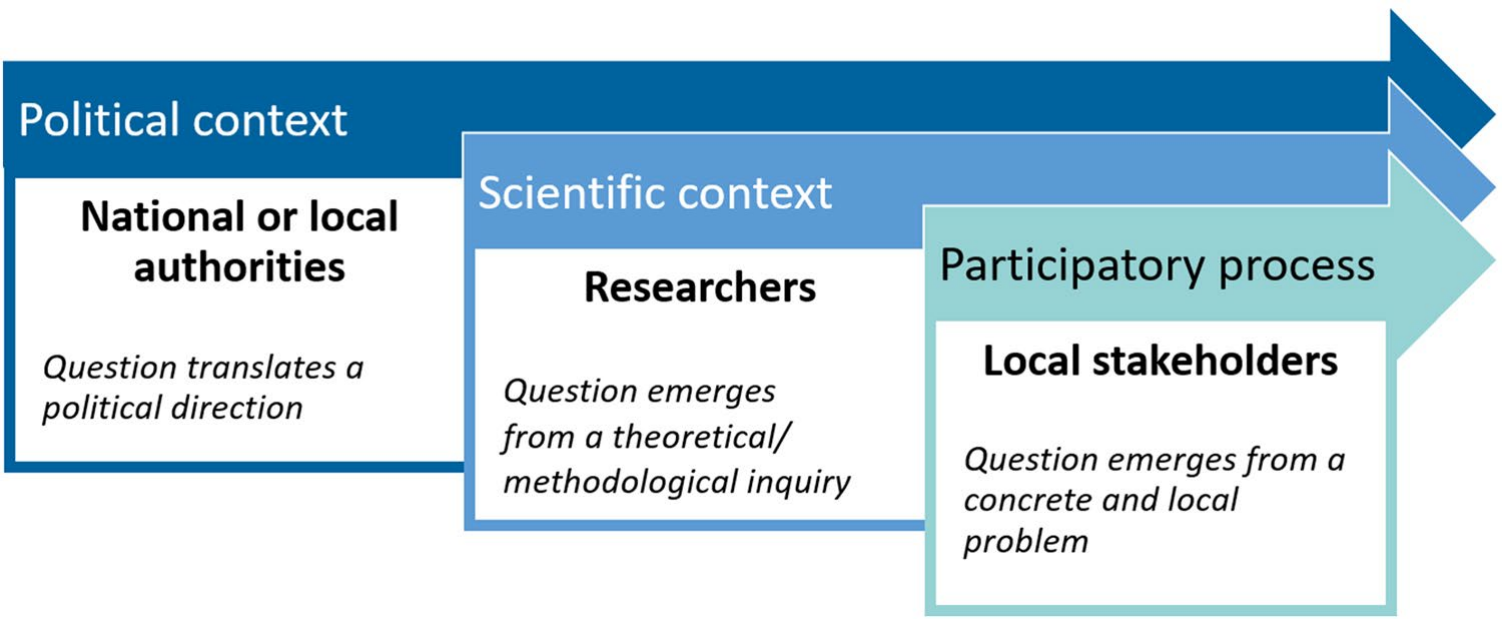
Diversity of contexts where a research problem can be established in participatory research

In this paper, we look more specifically into the participatory process of establishing a joint problem. We define a problem as Pearce and Ejderyan (2020) in the sense that a problem exists when a current state differs from a desired state. The foremost importance of establishing a joint problem for participatory research—when stakeholders and scientists jointly frame the problem at stake—has been repeatedly stated (Hirsch-Hadorn et al. 2008; Jahn et al. 2012; Norström et al. 2020; Steger et al. 2021). In practice, this process is rarely documented (Etienne, 2013) as framing the problem itself is mentioned as an important step but the description of real processes are missing (Pearce and Ejderyan 2020). Some rare papers detail the process but the authors themselves mention that the process remains a “consulting rather than participatory” one (Schäfer and Kröger 2016). In practice, most of these research processes are more often initiated by external institutions that are not local stakeholders (research institutions, NGOs, international organizations, states and other public institutions) (Voinov and Bousquet 2010). In particular, the share of power over the framing of the research is critical (Fritz and Meinherz 2020). In that sense, we can distinguish three types of arenas where problems can be framed (Fig. 1). First, cases where the problem is framed by political authorities and thus researchers accompany a political change (Bourgoin and Castella 2011); second, when researchers themselves have the control to make the problem fit with their conceptual or methodological objectives (Houet et al. 2017; Sun and Müller 2013); finally, a third type of case when problems emerge along with participatory processes regarding concrete and local problems, whether at the initial stage of the research (Reed et al. 2013) or through iterative modelling loops (Barnaud et al. 2007; Anselme et al. 2010; Luthe 2017).

The risk of missing out on joint problem formulation is that power asymmetries between external institutions (in general holding the initiative and budget) and local stakeholders lead to overshadowing local problems. To overcome this issue, the challenge is to know how to connect an externally constructed problem with local problems. While the process of structuring a problem in a participatory fashion—once it has been set—has been explored for many years (Etienne et al. 2011; Rosenhead and Mingers, 2001; Shaw et al. 2006), methods to jointly frame the problem itself have not been described much in the literature. Even in very integrative participatory processes where scientists, project initiators and local stakeholders are well connected, the co-construction of a joint problem remains mostly undescribed (Campo et al. 2010; Gaddis et al. 2010). This suggests that project initiators usually hold the power over the decision about how to frame the problem at the beginning. Even though some power asymmetries can be overcome during the process (Barnaud et al. 2010), the importance of setting the initial problem remains significant (Etienne 2013; Grimm and Railsback 2005) and probably conditions the overall direction of the research process towards the initiator’s interests rather than those of local stakeholders.

The co-construction of the initial problem is particularly challenging when local stakeholders’ concerns are not aligned with those of the initiators (Lamine 2018). Such a situation can arise when different parties hold different worldviews. For example, despite a wide consensus among scientists about threats from climate change there are enduring uncertainties, and citizens may remain sceptical about their significance (Whitmarsh 2011), probably as values and political allegiances often overshadow facts about the topic (Hornsey et al. 2016; Milfont et al. 2021). Prioritization on this specific matter has already led to intense controversies (Lomborg 2003; Pielke Jr 2004). The difficulty to co-construct a common problem can also come from psychological distance, when a decision needs to be made now for a distant future impact (Liberman and Trope 1998; McDonald et al. 2015). Moreover, even local stakeholders may hold very diverse mental models about a similar SES (Mathevet et al. 2011) and challenge the possibility to come to an agreement on the most important matter locally. Even though such ambiguity (or social uncertainty) between stakeholders can be handled or even elicited (Brugnach et al. 2011; Salliou et al. 2017), uncertainties in general are thought to hinder decision-making. It has been shown, for example, that the decision to act collectively to avoid a climate change tipping point was significantly reduced by uncertainties about the threshold temperature triggering it (Barrett and Dannenberg 2013). Finally, it may simply be the case that there is no complex joint problem to tackle requiring a participatory approach. Indeed, not all joint problems might require a participatory process. According to the post-normal framework from Funtowicz and Ravetz (1994), participatory approaches are relevant when relying on experts and normal science is not sufficient to solve a problem. This happens when uncertainties and stakes are high. However, it is not always clear when a given system is in such a situation.

Consequently, better knowing how to connect the interests of local stakeholder with those of project initiator in participatory research processes is required for initiating a process. We did so in a research project where our main research question related to adaptation pathways (Wise et al. 2014) in the context of global change in mountain socio-ecological systems (SES). In particular, we were interested in the capacities of local communities to mobilize ecosystem services for their climate change adaptation (Lavorel et al. 2019). SES are defined by the complex interactions between humans, their institutions and ecosystems (Ostrom 2009). This project was conducted in parallel at two sites, in the Southern French Alps and in the Swiss upper Valais. Mountains are particularly interesting for climate and adaptation scientists, because the impact of climate change is particularly strong, e.g. melting glaciers, snow reduction challenging tourism activities and rising natural risks like avalanches and landslides (Klein et al. 2019). With our research questions framing the initial problem and together with local stakeholders, we co-designed a board game called “GAME OF CRUXES” (a crux is a difficult section in a climbing or mountaineering route). Through visioning workshops with local stakeholders and conceptualization with relevant local and scientific experts, we created a board game including local understanding of important dynamics of these mountain SES. In this paper, we analyse whether this type of game and inclusive co-design enabled stakeholders and scientists to identify potential joint problems for participatory research.

## Methods

This section describes how we designed the game with the objective to support scientists and stakeholders to identify joint problems for participatory research.

### Overall game design approach

To design such a game, we applied two approaches, namely companion modelling (Etienne 2013) and backcasting (Robinson 2003).

Companion modelling specializes in participatory modelling, notably with the creation of games and simulations to generate interactions between stakeholders. This approach has been used successfully to create many serious games over the years. Even though the scope of this method is originally about natural resource management, its use has extended to other topics like urban planning or risk management. In this approach, the game is usually a means to an end: the discussion and learning triggered by the interactions of players during the game session and particularly in the post-game debriefing session. In this paper, we mobilize three key steps from the companion modelling approach to design our game: (1) establishing a common conceptualization of the SES system at stake with key stakeholders, (2) translating main concepts and interactions from the previous step into game mechanics, (3) facilitating and observing game sessions involving a diversity of stakeholders together with a post-game debriefing to reflect on the experiential learning.

Prior to companion modelling, we used a visioning exercise with stakeholders, inspired by the backcasting method (Robinson 2003). This method leads to the production of a normative targeted future with participating stakeholders. Diversity of participants is thus essential to guarantee that the vision is as legitimate as possible. In this process, scientists can introduce and thus suggest their topic of interest to sub-groups of stakeholders. Subsequently, with scientists as facilitators (and thus neutral at this point), stakeholders list the most important elements they wish for the future of their region. The analysis of the stakeholders’ vision and scientific input can thus provide some insights about potential overlapping interests. The visioning workshop is conducted before the main steps of the participatory modelling process described above, as this step gives the most freedom for suggestions by stakeholders. In most of the modelling process, it is advisable to start from a wide consideration of descriptive elements before slowly tuning and simplifying the model (Edmonds and Moss 2005). The vision provides a key story telling component to involve players in the serious game (Mitgutsch and Alvarado 2012; Mildner and Mueller 2016).

### Common conceptualization of the socio-ecological system

While different conceptualization tools exist, like rich picture or fuzzy cognitive mapping (Voinov et al. 2018), we opted for the ARDI (Actor, Resources, Dynamics, Interactions) method developed by members of the companion modelling community (Etienne et al. 2011). The ARDI method was designed for participatory conceptualization of socio-ecological systems. Stakeholders discuss and agree in workshops on significant agents, objects and their interactions. This method has been commonly used as a preliminary step towards the construction of serious games (Etienne 2013). The originality in our use of this approach is to integrate members of the scientific team as stakeholders in the co-construction of the conceptual model. Doing so, the scientific representation of the SES from project members are represented and integrated in the future game alongside those of other stakeholders.

### Translating main concepts in game mechanics and overall principles for game design

A conceptualization like ARDI allows to identify key actors and resources. Typically, from an ARDI conceptualization, several translations to a game are possible: (1) the scale of resources may give an indication on the main scale of the game, (2) the time scale of key dynamics may give indication on the time step in the game, (3) actors with significant acting power on the system can be translated into players, and interactions where they are involved turn into actions in the game, (4) conflicts in actions (e.g. two different actors using the same limited resource) may form the core of the game mechanics. (5) Thematic clustering, i.e. several interconnected resources and actors, suggests a particularly complex part of the system that might be especially relevant to include in the game.

Apart from these few principles, the game design itself is more art than science as it involves a creative process. This creative process is enhanced by practice, knowledge and examination of other games, serious or not, for inspiration and guidance (Mildner and Mueller 2016). In general, it is advised to create and tune a game so to ensure a good flow, not too hard and frustrating and not too easy and boring (Csikszentmihalyi and Csikzentmihaly 1990). Game testing and iterative loops of game design is thus essential (Macklin and Sharp 2016). For a serious game, game testing with domain experts is essential to keep the factual content in line with the described system (Mildner and Mueller 2016). Additionally, good narrative as well as good aesthetics enhance player’s engagement (Mitgutsch and Alvarado 2012). To guarantee the narrative quality, visioning workshops aim at describing a “desirable future” (Myers and Kitsuse 2000; Brondizio et al. 2019) and thus guarantee a good level of engagement as the game explores the potential futures of the place these stakeholders live in. Players should be able through their actions to implement elements of their vision during the game.

### Prepare an observation and debriefing protocol

A key part of any serious game development should include a prepared in-game observation and post-game debriefing protocol (Hassenforder et al. 2020). For a role-playing game or board-game, these steps often rely on scoring sheets for observers with pre-established indicators to be collected by scientists during the game session. For a game aiming at identifying potential joint problems, the protocol should include both (1) which topics generate the most interest from stakeholders during the game and (2) how the elements of interest from the scientific team are mobilized (or not) during the game sessions. Logically, these observational elements are used in the post-game debriefing to openly discuss the potential for joint problems between game players and research team scientists.

## Results

**Fig. 2:**
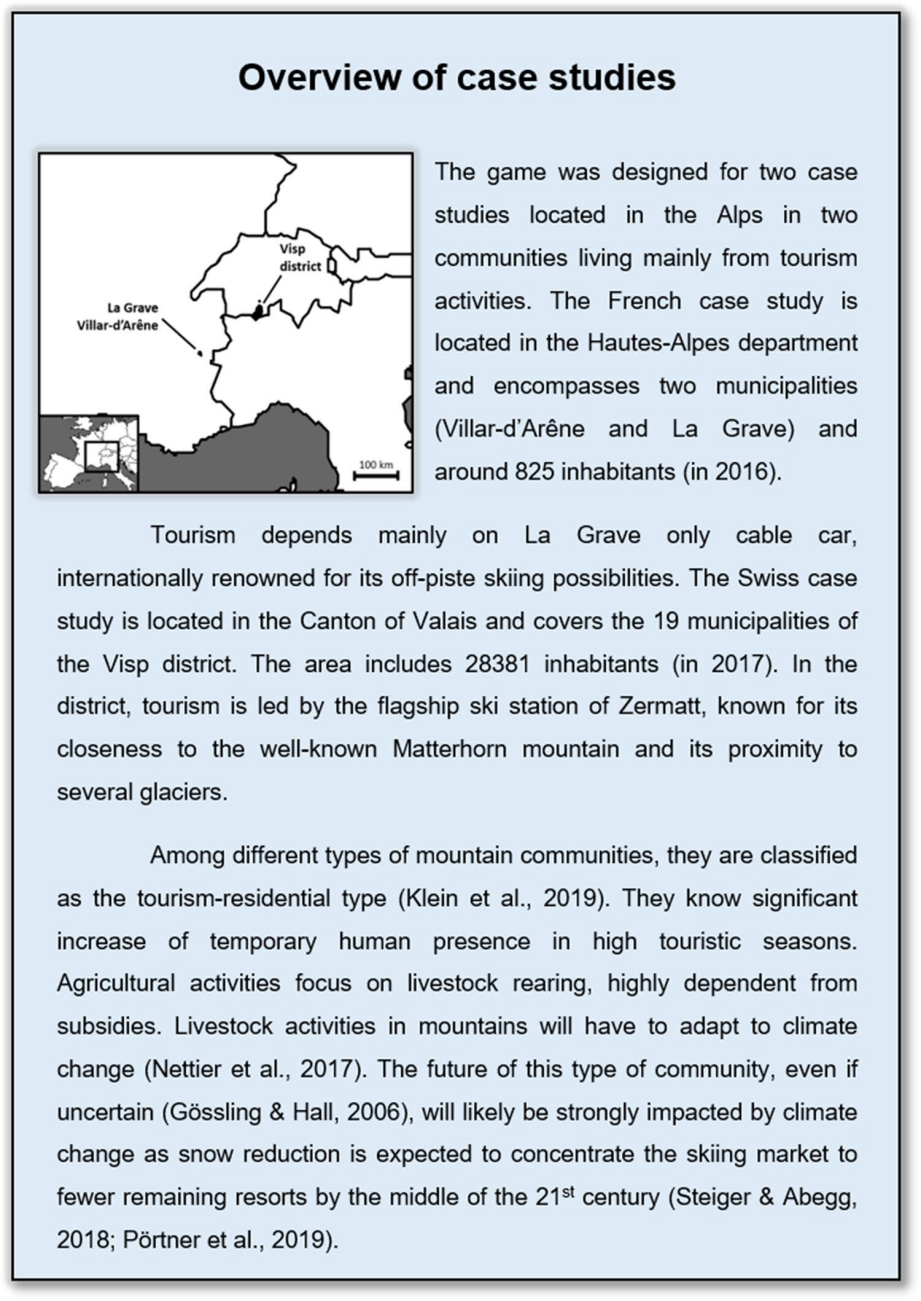
Presentation of case studies

In this section, we describe three different levels of results. First we describe the specific process of game design introduced in the Methods section for our two case studies (see Fig. 2). Secondly, we provide results on the capacity of the game to cover topics of interest for participants. Finally, we detail our analysis of the observation and debriefing of game sessions leading to: (1) the ranking of key issues and (2) the potential identification of joint problems between scientists and stakeholders.

### Game co-design

In this section, we present the main steps of the game co-design as described in the Methods section. We detail the visioning, the conceptualization and the board game design processes. For more details of the game itself -appearance and overview of rules- see Online Appendix 1.

To co-design the game, we first organized and facilitated workshops with local stakeholders in both case studies during which stakeholders defined a collective vision for 2040. We set the end date of the backcasting to 2040 as a compromise between a very long-term perspective (where psychological distance to individuals and climate change impact would be high) and a short-term perspective (little psychological distance and room to discuss the future). At the time of the design, 2040 was also the time when different climate scenarios started to diverge (van Vuuren et al. 2011). Before the construction of the visions, the research team presented their perspectives about climate change and adaptation to inform stakeholders of the objectives of the scientific project. Visioning workshops were based on mixed techniques (focus groups together with drawing, writing and participatory mapping). Our scientific team facilitated both French and Swiss visioning workshops but were not included as stakeholders. Eleven stakeholders were involved for this exercise in Switzerland and 45 in France. We invited stakeholders with the intent to balance major sectors and diverse scales of action. Building a vision for each site was done in sub-groups of four to five people and was not limited regarding the array of topics that could be included. In Switzerland, a graphic designer helped each sub-group to build their vision. Summaries of the Swiss and French visions are presented in Table 1. After the workshops, our research team compiled the main elements of each vision in a synthesis document shared with stakeholders.

**Table 1:** Description of main topics in the visions for 2040 developed with stakeholders at both study sites (La Grave Villar-d’Arêne for France and Visp district for Switzerland)

Vision for 2040	La Grave and Villar-d’Arêne, France	Visp district, Switzerland
Tourism	Focus on tourism requiring limited investment in infrastructures (contemplation, learning experiences etc). Favouring longer stays	Increase in high quality accommodation and limited empty dwellings. Development of sustainable tourism through education of tourism workers. Optimization of railways connecting valleys and supporting year-round tourism
Landscape	Maintain current landscape, at least partly	Agriculture maintains the landscape open. Old buildings are renovated. Preservation of local forests
Agriculture	Agriculture is strongly linked to tourism and local consumption. Local production is diversified, processed locally and exchanged regionally between short supply chains	Regional agriculture is diversified and supplies seasonal needs in valleys. Traditional agriculture is part of the tourist experience.
Forestry	Source of firewood	Increased local use of regional wood, promoting local identity, jobs and green buildings.
Mobility	Shared modes of transportation as well as a greenway between villages and hamlets.	Fast, secure and environmentally responsible accessibility to and from the valleys. Autonomous vehicles and e-mobility
Settlement	Second homes are permanently occupied. Urban sprawl is marginal. Waste is valorized locally. Internet access as in urban areas	Preserved and modernized villages. More digitalized environment increases self-employed workers’ presence in the valleys
Demography	Higher proportion of residents	Growing population. Higher proportion of residents and temporary workers
Energy	Hydropower and solar energy	Hydropower and solar energy
Economy	Short supply chains	Label for local crafts and regional products
Governance	Rich social fabric, sharing, solidarity	Saas and Matter valleys jointly manage their development and make key decisions together
Vision for 2040	La Grave and Villar-d’Arêne, France	Visp district, Switzerland
Tourism	Focus on tourism requiring limited investment in infrastructures (contemplation, learning experiences etc). Favouring longer stays	Increase in high quality accommodation and limited empty dwellings. Development of sustainable tourism through education of tourism workers. Optimization of railways connecting valleys and supporting year-round tourism
Landscape	Maintain current landscape, at least partly	Agriculture maintains the landscape open. Old buildings are renovated. Preservation of local forests
Agriculture	Agriculture is strongly linked to tourism and local consumption. Local production is diversified, processed locally and exchanged regionally between short supply chains	Regional agriculture is diversified and supplies seasonal needs in valleys. Traditional agriculture is part of the tourist experience.
Forestry	Source of firewood	Increased local use of regional wood, promoting local identity, jobs and green buildings
Mobility	Shared modes of transportation as well as a greenway between villages and hamlets.	Fast, secure and environmentally responsible accessibility to and from the valleys. Autonomous vehicles and e-mobility
Settlement	Second homes are permanently occupied. Urban sprawl is marginal. Waste is valorized locally. Internet access as in urban areas	Preserved and modernized villages. More digitalized environment increases self-employed workers’ presence in the valleys
Demography	Higher proportion of residents	Growing population. Higher proportion of residents and temporary workers
Energy	Hydropower and solar energy	Hydropower and solar energy
Economy	Short supply chains	Label for local crafts and regional products
Governance	Rich social fabric, sharing, solidarity	Saas and Matter valleys jointly manage their development and make key decisions together

**Fig. 3:**
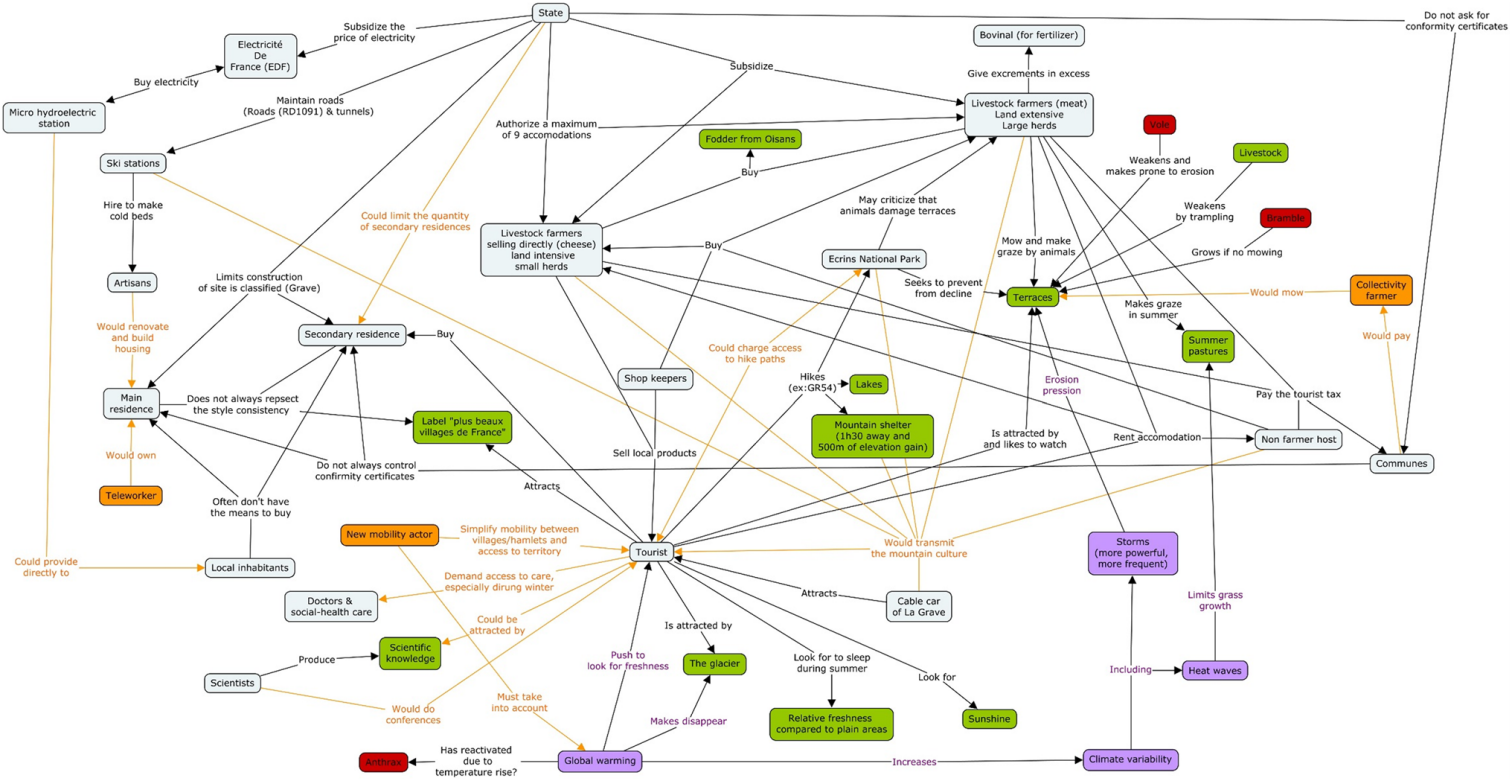
Participatory conceptualization of the socio-ecological system of the Pays de la Meije (France). Light blue boxes indicate an actor. Green boxes indicate a resource. Red boxes indicate an ecosystem disservice. Purple boxes refer to climate change. Orange boxes indicate potential new actors and interactions in the future

As a second step, we organized an ARDI workshop in the French case study to build a conceptual model of the SES (Fig. 3). The workshop involved five experts covering a diversity of academic and local stakeholder perspectives. These experts are part of a core group that participated in all stages of the process. The group, four men and one woman, included: a retired cattle breeder, elected municipal official and owner of a tourism business; the communication manager of the cable car company of La Grave and elected municipal official; a senior scientist from our research team anchored on the site and specialized in ecology; the scientific manager of the Ecrins National Park; and the climate plan coordinator for the Briançonnais council of municipalities (Pays du Briançonnais). Interestingly, while visions (which did not include scientist perspectives) did not incorporate any reference to climate change events, the conceptualization did include this important phenomenon from our team perspective (in purple in Fig. 3).

As a last step, and based on visioning and conceptualization, we designed a board game. First, we translated many elements of the vision into in-game possibilities (Table 2). We can distinguish three types of translation: (1) individual action to change one’s activities toward an element of the vision (e.g. become an artisan), (2) players grouping their resources to invest in a collective project (e.g. hydropower plant) using a typical public good game framework (Ledyard 2020), (3) take individual or collective decisions regarding tourist flows and local demographics (e.g. settle a new farmer). Thus, players could manipulate and influence the trajectory of these elements at the heart of their vision for the future. Second, we mobilized two key clusters of the conceptualization in the board game design: (1) dynamics centered on tourism and tourists (bottom and left part of the graph in Fig. 3, centered on the “tourist” actor and the “secondary residence”); and (2) dynamics centered on agriculture and the management of pastures and terraces (upper right part of the graph in Fig. 3, centered on “terraces” and “livestock farmers”). The game was finalized after testing by members of the research team to tune its flow and playability. A graphic designer helped to produce the final board game and improve its aesthetics for player’s engagement.

**Table 2:**
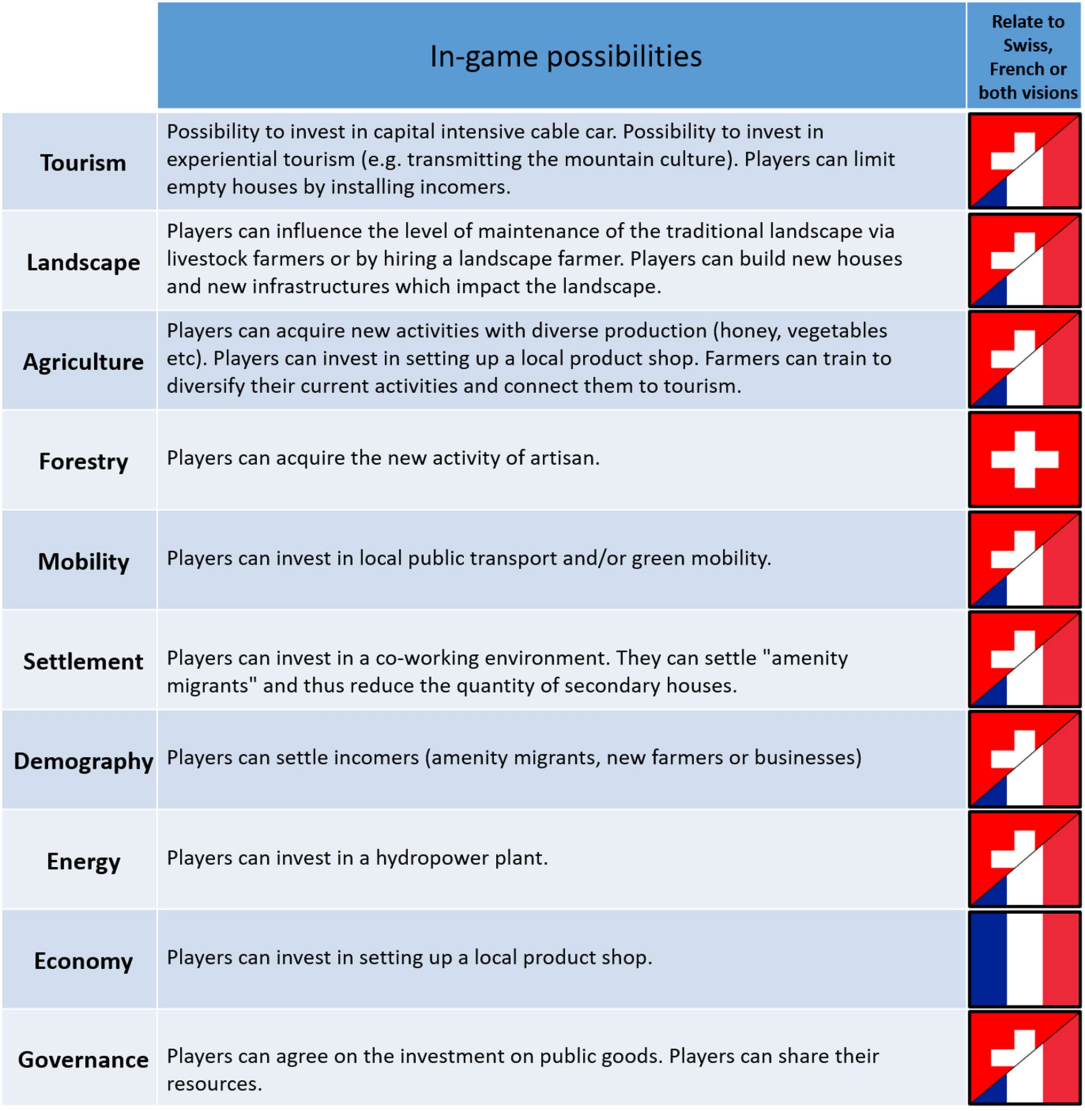
In-game mechanics related with main topics and correspondence with French, Swiss or both visions for 2040

Finally, an observation and debriefing protocol was put in place. For each game session in the French case study, at least one observer recorded main decisions, discussion points and actions taken during the game by players. We designed a one-hour debriefing to both (1) collect direct feedback from the game experience from players and (2) discuss the significance of ecosystem services to players for adapting to future changes and maintaining their presence on site in the future. The direct feedback (1) engaged players to reflect on the final state of the game board compared with the pre-established vision they had for their area by 2040. Facilitators from our team used this feedback to discuss critical barriers on the pathways to the vision. This step is consistent with the backcasting approach we followed. The discussion (2) was an intentional move from the scientific team to question players about the capacity of the SES to follow successful adaptation pathways which is the major topic of interest from our research team (Lavorel et al. 2019). In France, we invited all participants in the participatory modelling process (local inhabitants, co-designers and players) to a final workshop where we introduced and discussed together our learning from the game sessions.

We conducted ten game sessions in France involving 36 participants from a diversity of stakeholders. Table 3 describes the diversity of stakeholders and their real-world occupation. The scientist from our team who participated in the conceptualization phase as a stakeholder also played during one game session. Finally, we also tested the game developed for the French case study with some Swiss stakeholders with whom we also developed visions for 2040. As can be seen in Table 2, the commonality across the two sites gave us good reasons to think that the design of our game was able to cover most of the topics in Switzerland. This test was conducted with four scientific and public servants working at the cantonal level of Valais. We engaged with them based on their interest for such a game-based approach and willingness to engage in science and society partnership to solve potential problems. Through this game session, we tested the capacity of our game designed for the French case study to identify a joint problem for a different mountain area.

**Table 3:**
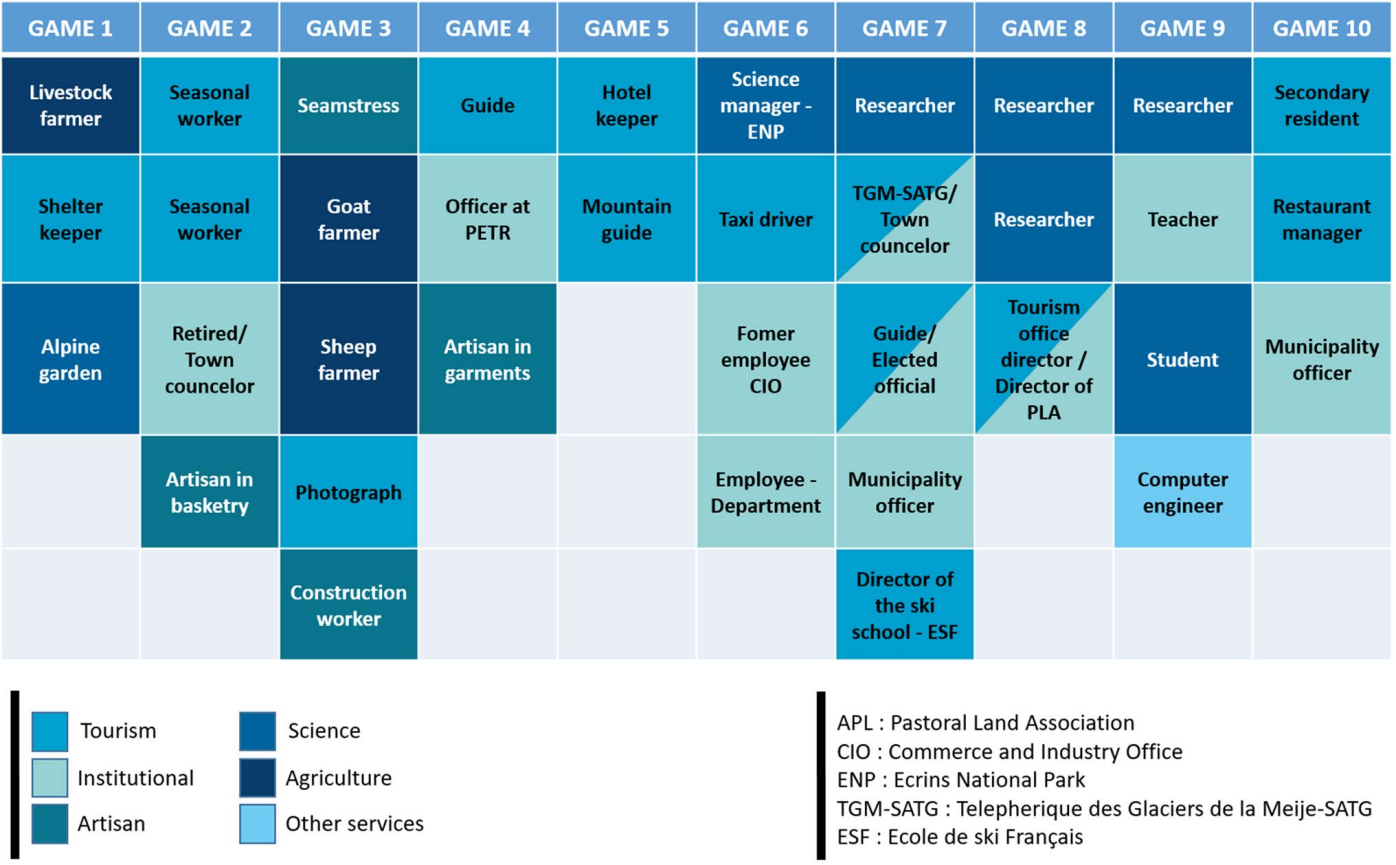
Description of participants in the game sessions at the French study site, color(s) refers to their sector(s) of activity

Color(s) refers to their sector(s) of activity

### A game covering main topics of interest

From our observation reports, we designed Table 4, which indicates the main topics of the vision for 2040 discussed between players during game sessions and/or the debriefing. All game sessions covered at least six of the ten topics of the vision. Four topics were discussed in all games: tourism, landscape, agriculture and economy. The topic of forestry was least discussed, which is quite logical considering that forest cover is scarce at the French site. Table 4 shows that the game was able to cover all topics of interest mentioned in the vision. Additionally, at both sites, players praised the game design for its capacity to capture the complexities of the dynamics of their mountain SES. Swiss players mentioned that the game properly depicted the main topics faced by mountain communities living from tourism and agriculture in Switzerland. This feedback is all the more interesting since the game was based on the conceptualization of the French case study (Fig. 3).

**Table 4:**
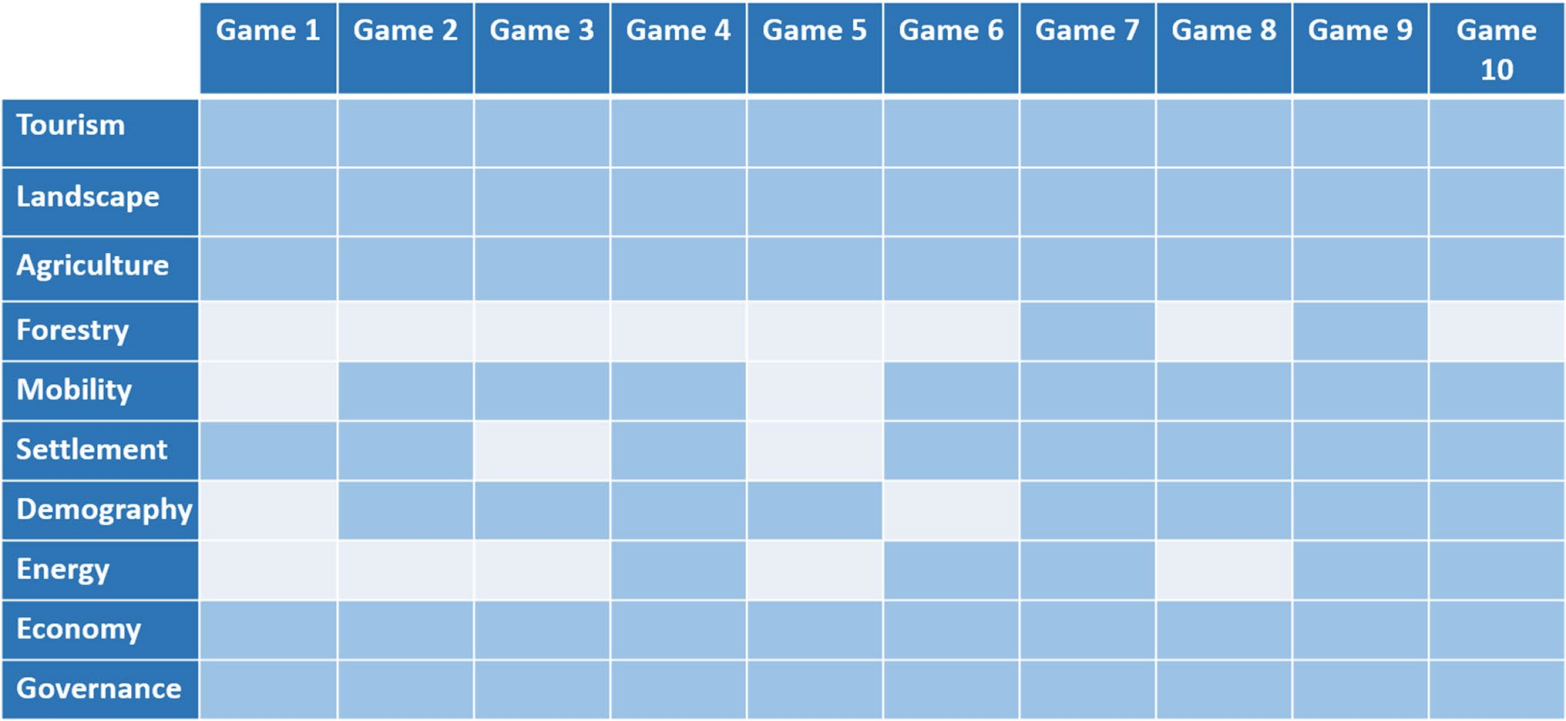
Topics of the vision for 2040 discussed by players in the French game sessions (dark blue indicates the topic was at least discussed once)

### Listing and ranking issues from game session debriefings

Main issues discussed by stakeholders in the French case study during game debriefings are summarized in Table 5. They are extracted from the observer’s report of each game session. The most striking element of this table is the issue regarding governance and power, with seven and five game sessions, respectively, mentioning collective action and decision-making as an issue. Our game design allows quite easy collective action and decision-making as there is only a handful of players, seeing each other face to face. As such, players were keen to indicate the discrepancy between the in-game experience and real-world difficulties. More precisely, throughout these debriefings, well-off local families as well as established institutions [National Park, Municipal councils, to a lesser degree the Pastoral Land Association (PLA)] were often depicted as hindering local development by blocking decisions, top-down decision-making or through regulations. While issues about power sharing at the municipality level is somehow logical for a political institution like a municipality, it is more surprising for the Écrins National Park (ENP). In five game sessions, the ENP was considered by all players as a constraint through the enforcement of regulations and restrictions (Table 5). The problem around ENP is all the more critical as it was indirectly connected by players with the issue of wolf impact on farmer livelihoods, also mentioned during three game debriefings (Table 5). However, the wolf issue is not specific to the case study. As one player put it: “*it is a national drama in the countryside and matter of applause in cities*”. Indeed, to many farmers, the ENP is favorable to the wolf’s presence, which goes against their interests and is consistent with an urban mindset. A final workshop, attended by the director of the ENP, confirmed the existence of a gap between inhabitants and the park. This situation shows a clear need for concertation to move eventually from a tense, conflictual situation into a more collaborative state.

**Table 5:** Main issues discussed during debriefing sessions with game participants

Problematic	Game sessions mentioning the problem	Summary of the problematic
Collective action	7	While collective action is made easy in the game, players mentioned the difficulty to get similar outcome in reality. In particular, the opposition of the two neighbouring villages makes difficult the possibility to group them politically and even to decide on common issues. Some players mentioned the power of a few established families and institutions (like the Pastoral Land Association) to lock this collective action potential
Local decision making	6	Decision making at the municipality level is externally considered as a closed and top-down system with limited movement among elected officials and not inclined towards participation, communication or concertation. Internally, this institution is hindered by the lack of power over private actors and voters’ absenteeism in the area (secondary house owners can often vote)
Écrins National Park	5	A constraining institution, hindering local development through its regulations. The institution is considered to have the power to federate stakeholders but a rupture of dialogue is mentioned with livestock farmers
Agriculture	4	The role of subsidies is mentioned as negative and pushing farmers out of local development considerations. The role of pastoralism and transhumance could be further discussed and redefined, especially in the Pastoral Land Association managing pastures
Wolf	3	The controversial presence of wolves in the area, challenging livestock farmers’ livelihood and contributing to closing the landscape
Tourism	3	The local tourism model in general and more specifically the organization of the tourism office and the cable car company are questioned
Social	3	The locking power of a few wealthy families. Separately, the need for new population and their integration is mentioned
Economy	2	Difficulty to finance collective projects

The game sessions also indirectly shed light on in-game elements relating to the mountain SES, which were almost not discussed during debriefing sessions. In particular, the game rules clearly allowed players to settle new people permanently, to build new houses or manage hotels. While these topics of settlements and demography appear in both French and Swiss visions for 2040 (Table 1) and in the French conceptualization model (Fig. 3), it did not stand out as a key problem during debriefing sessions. Finally, climate change and adaptation were not seen as a key problem even though we incorporated some external events in the game like climate events, natural hazards or an oil crisis.

### Identifying joint problems between scientists and local stakeholders

**Fig. 4:**
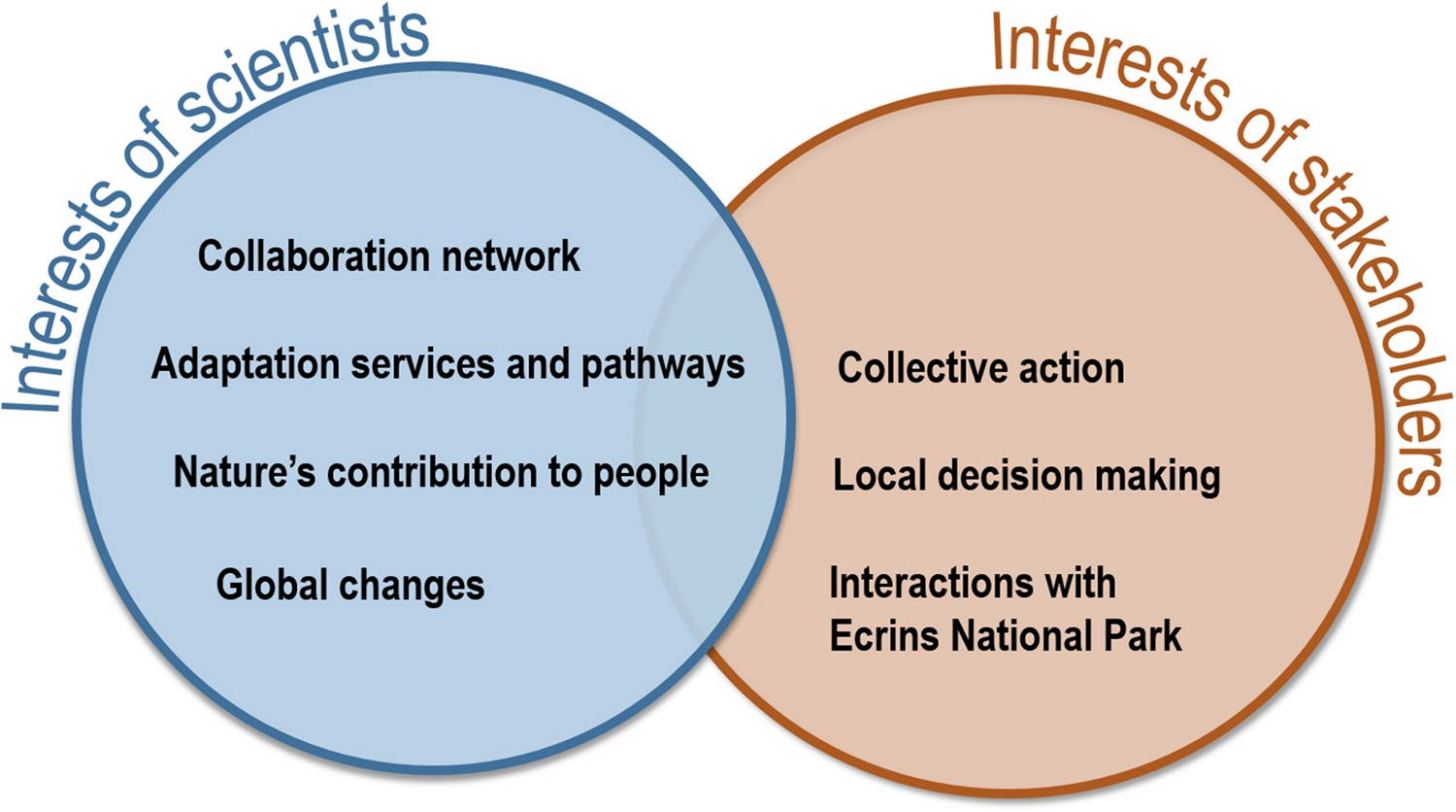
Main issues for scientific and local stakeholders from the French study site

We sum up in Fig. 4 the current state of issues of interest from our side as scientists and the main ones from the perspectives of local stakeholders in the French case study. As mentioned in the previous section, game sessions were useful in revealing local stakeholders’ main issues by proposing a game experience with a wide diversity of topics. Thus, we could evaluate how scientific and local stakeholder interests could eventually overlap for further participatory research. As we show in Fig. 4, both spheres of interests do not overlap much. Even though the scientific frameworks used by our scientific team about adaptation pathways (Lavorel et al. 2019) and nature’s contribution to people (Díaz et al. 2018) include the importance of local decision-making and collective action, they had a limited overlap with stakeholders concerns. The potential for bridging both parties is not straightforward, as issues mentioned by stakeholders related more with political sciences, which is not the core discipline of our scientific team (ecology, agronomy, geography, landscape planning, sustainability science). Future participatory research could consider involving scientists holding such knowledge.

In the Swiss case study, the debriefing session mainly led to two conclusions with game participants: (1) the game experience was efficient in capturing most of the main features of a tourism-residential mountain SES and is an engaging device for discussion, and (2) according to players—who are also potential partners for further participatory research—no problem was complex enough to legitimate the organization of new gaming sessions. The risk was clearly stated by these partners that they might lose credibility by proposing such workshops with local stakeholders. Without a problem complex enough to require a participatory process, it would be difficult to legitimize further joint research and mobilize local stakeholders. As a consequence, no further game sessions were considered.

To conclude this results’ section, we found that the game experience was useful to assess the potential for joint problems. In our two case studies, we found no joint problems justifying further participatory research with our specific research team. In France, we identified two complex problems with high stakes and uncertainties, as described above, but they hardly overlap the core interests of our research team. Additionally, these joint problems would require expertise that was not present in our research team. In Switzerland, identified issues were not considered to represent sufficiently high stakes by the game participants and our research team to justify a further participatory process. Despite these difficulties to set a joint problem, the process of openly explore the space of potential collaboration was appreciated by all the parties, and the gaming approach was praised for its inclusiveness, transparency and capacity to generate a unique space of dialogue between diverse parties.

## Discussion

### The balance of power between scientists and other stakeholders

All along the process, our research team held a significant amount of power over many decisions about game development. Even if we had full control on techniques that were used in the process, we also used techniques that limit this power. Notably, visions were built entirely on stakeholder’s ideas and wishes. Even when the research team provided some prior background information about climate change and adaptation to participants, that appeared to play no role in influencing the following exercise as visions in both Switzerland and France do not explicitly include anything about these topics. As described in the Results section, during the conceptualization phase, we decided that scientists from our team were significant stakeholders that could not be ignored in describing how the SES works, and one senior scientist was thus included. In this workshop, this scientific stakeholder was one out of five individuals who were not part of the research team and this seemed to be enough as a counter-power. The rest of the team remained at all other times as neutral as possible, following a “critical companion” posture (Barnaud and Van Paassen 2013) in facilitation and observational roles. We believe that the process described in this paper is enough to limit scientist power on defining the joint problem, the proof being that, contrary to our research objectives, no joint problem justifying a participatory process was clearly accessible to us. For a research project that would emerge from a political context rather than from scientists (Fig. 1), a similar transparency about main topics of interest from the political agenda would yield a similar limitation of influence on the game design and outcome.

### The use of games to assess complex joint problems requiring participatory research

In this paper, we show how games designed by scientists with stakeholders can be used as a tool to identify joint problems. To do so, we designed the game together with stakeholders by encompassing a wide array of interconnected topics. As participants experience this diversity in the game, it is then possible to discuss in the following post-game debriefing which of these topics are complex problems. We even found that game sessions could sometimes identify complex problems that were not present in the game, nor in the game design process. This showed the specific usefulness of the post-game debriefing compared to the sole listing of issues from a vision or a conceptualization exercise.

However, it is important to note that our game design process described here can only demonstrates its capacity to identify the absence of a complex joint problem. As many practitioners of participatory processes are aware of, participatory research does not start from a well-established problem-framing that would legitimize with certainty an intervention and the mobilization of stakeholder’s time (Lang et al. 2012). The risk of the “tyranny” of participation is never far (Cooke et al. 2001). The lack of post-normal issues, with high stakes and uncertainty, may be one explanation of regularly observed “stakeholder fatigue”, even though other explanations like poor communication are also possible (Jönsson and Swartling 2014; Bracken et al. 2015). Even though using such a game does not come cheap (Barreteau et al. 2014), especially as funding is usually an issue at the initial stage of a participatory project (Luthe 2017), we suggest that designing a game to assess complex joint problems is worth the investment. Indeed, it can save incredible amount of stakeholder and researcher time and resources in the long-term by not mobilizing them when there is no need for it. In that sense, this game not only assesses the existence of a joint problem but allows, through game debriefing with stakeholders, to assess whether some problems are in the post-normal sphere legitimizing participatory research. However, the assessment that a situation is in such a post-normal frame is not well defined apart from listening to stakeholders’ feedback. The process to evaluate more systematically if a situation is with high stakes and high uncertainty remains to be better defined.

Additionally, because the tool is quite broad regarding the issues it encompasses, it could be potentially used in various mountain SES. The reusability of such a game could potentially save many of the initial development costs (Murray-Smith 2012). We have shown in this article how we were able to use this game to assess joint problems within a case study (in Switzerland) that was not the original SES used for the game design. In that case, it proved to be useful because it led to the agreement with our potential research partners that there was no known complex joint problem for participatory research. This potential for reusability in other similar SES can potentially save game development costs while getting the benefit of a joint problem identification assessment. We acknowledge that a single game session with another case study is not a strong validation for geographical transferability. Its potential for reusability across other mountain SES could be assessed through further trials.

### Limitations of the game

Overall, from our experience, the use of this game for joint problem identification remains limited by three main factors. The first factor relates to the presence of stakeholders around the board game. This device is situational and can only identify joint problems with the players who actually participate in the game. This can be a serious limitation as various stakeholders may have unequal capacities for participation depending on their wealth, education and political power (Agrawal and Gupta 2005; Barnaud et al. 2010). The second factor is the specific type of SES that is covered by the game, which Klein et al. (2019) call the “tourism-residential” type. The game, in its current state, would unlikely be directly transferable to other types of mountain SES mainly living, for example, of pastoral activities. Similarly, the use of the game for the tourism-logging type, close to the tourism-residential type from Klein et al. (2019) in the current form of the game, would probably require the addition of a logging module as this economic activity is not covered in the game. The third limitation is the cost attached to making such a game accessible to a wider public. Even if some game libraries exist online,^1^ they usually do not make the game itself available for autonomous use nor do they always describe what can be achieved with it, thus limiting their reusability. We advocate an open and participatory online library where all game designers could make their game available (print and play, models, etc.) and easily accessible for other modelers, game designers and scientists. Accessibility could be enhanced by digitalizing such games, making it accessible worldwide. Some online platform support the creation of virtual board game, like tabletopia or tabletop simulator. This option can be particularly helpful in COVID-19 times when face-to-face meetings are difficult. The capacity of digital serious board game to reach similar engagement with stakeholders is an open question.

Finally, a last limitation of this approach of using a game to identify joint problems is its novelty and the fact that joint problem assessments in practice are not well documented in the scientific literature. Thus, it is difficult to assess its efficiency. It might be interesting in a near future to benchmark different techniques in their cost and efficiency. A promising comparison with non-game-based approach could include the Social Multi-Criteria Analysis approach, which has similar objectives and also works in the framework of post-normal science (Munda 2004).

## Conclusion

Research connecting science and society requires the identification of a joint problem between both parties. The use of a game co-designed with stakeholders covering a wide spectrum of topics enables the creation of a space for exploring them, ranking their local importance and eventually identifying a concrete and complex problem requiring participatory research. We successfully designed such a game concerning mountain socio-ecological systems living mainly from tourism. Such games hold great potential for cost-saving, as they may help in revealing the presence or absence of a joint problem and the subsequent necessity (or lack thereof) to conduct a participatory process. They are also valuable for clearly identify the problem to be tackled between scientists and stakeholders from the start. Such a game holds some potential of re-use for similar socio-ecological systems.

## Supplementary Information

Below is the link to the electronic supplementary material.

Supplementary file1 (DOCX 2014 KB)

## Footnotes

1 For example: https://games4sustainability.org/gamepedia/, https://www.commod.org/modeles, https://guides.lib.umich.edu/c.php?g=282989&p=5955091.
