# Supplementary material for: Game of Cruxes: co-designing a game for scientists and stakeholders for identifying joint problems
Source: Sustain Sci. 2021 Jun 10;16(5):1563–78. doi: 10.1007/s11625-021-00983-2 (PMC8191445; doi:10.1007/s11625-021-00983-2)
Supplement: Supplementary file 1 — Supplementary file1 (DOCX 2014 KB) [file 11625_2021_983_MOESM1_ESM.docx]

### Appendix 1: General description of the game

Overview

The game is for four players playing each two inhabitants from a same family. The game still works from two to eight players by redistributing family members accordingly. All the players live in the same valley. Each game session involves five turns and each turn covers a period of four years, from 2020 to 2040. A game session typically lasts one hour and half.
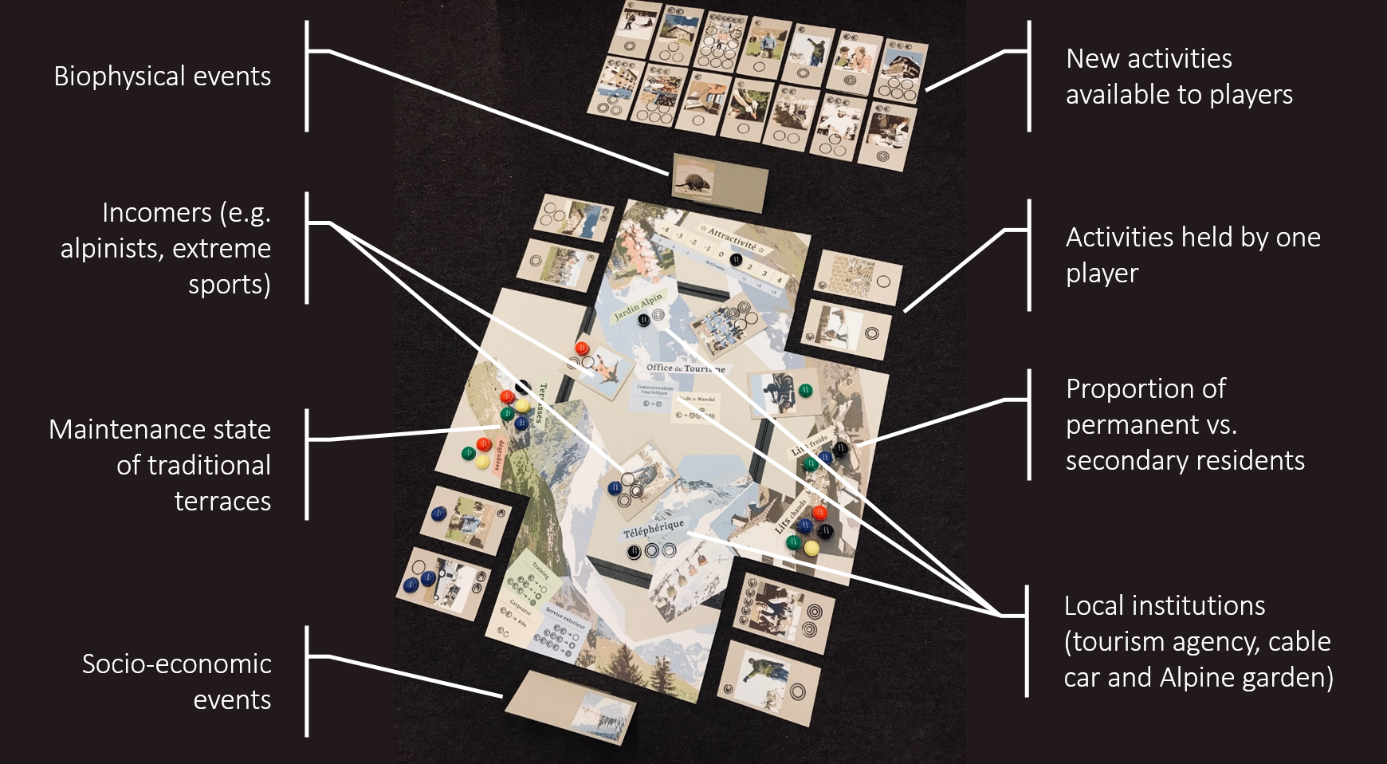


Players are inhabitants who try to maintain their presence by satisfying the needs through “incomers” (mostly tourists). Needs of “incomers” are an adapted three-level Maslow pyramid with basic needs, recreational needs and self-actualization needs. In the game, each family member is originally assigned a business (farmer, hotel manager, mountain guide etc.) and a bit of capital. Through their activities, players/inhabitants can fulfill the incomers’ needs by providing the demanded services (e.g. a hotel satisfies the basic need of accommodation) and thus obtain an income to cover their living costs. Key local institutions can also provide services to incomers if players wish doing so. Costs, incomes and flows of incomers were not based on or made proportional to real data but designed to create a feeling of realism for players. For example, each turn farmers receive one monetary unit as income from their livestock and one monetary unit as subsidies from the European Union Common Agricultural Policy. Such parametrization is far from real data but conveys the reality of farmer’s income dependency.

Actions for players

At the end of each turn, incomes earned by players are deducted from the costs to sustain their professional activities and presence in the area. Players can also perform several other actions available each turn: (1) diversify existing activities, (2) change activity, (3) invest in housing or (4) invest in collective projects. Investing or changing an activity means that the player, by training or diversification, modifies the quantity or quality of the service(s) potentially provided to incomers. Investing in collective action is strongly rooted in the vision developed by local stakeholders in the workshops beforehand. Most of the available collective projects originate from the visions established by stakeholders and are designed as a public good game. This game mechanics creates difficult trade-off between individual (diversifying or change one’s activity, invest in housing) and collective investments for players.

Biophysical and social events


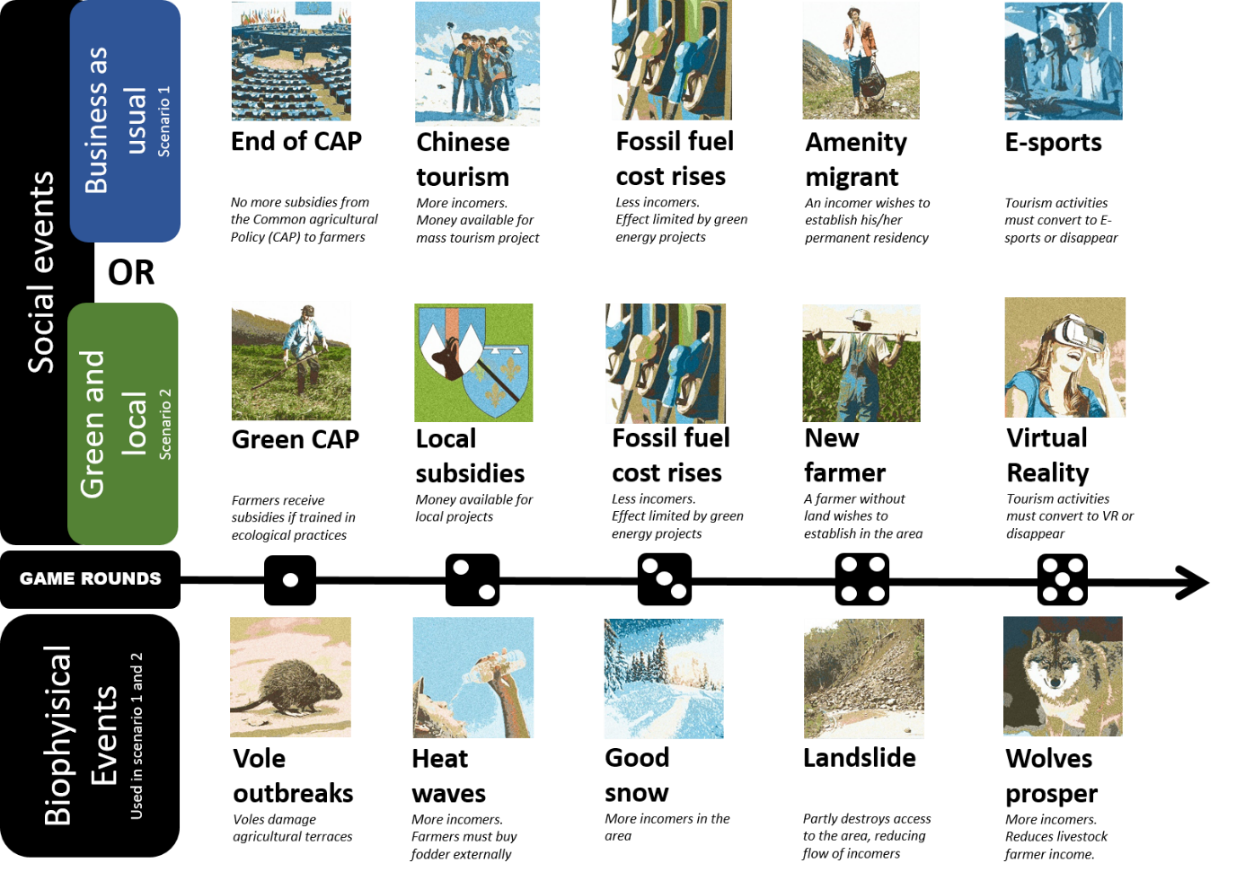


Overview of events used during the five rounds of a game session

Each game round involves one socio-economic as well as one biophysical event. We designed these events to provoke players to react to global change as well as nature’s contribution to people in the area. Two sets of events were designed to create two different scenarios: a “business as usual” scenario and a “green and local” scenario (See figure above). Each scenario includes social events in the same turn order about agriculture, economic development, energy, migration and technology. We used them to trigger potentially some discussion about these topics.
